# Growth, yield and economic performance of lettuce (*Lactuca sativa* L.) in response to nitrogen and phosphorus fertilization under irrigation in Bahir Dar, Ethiopia

**DOI:** 10.1038/s41598-026-47274-3

**Published:** 2026-04-09

**Authors:** Sefinew Shibabaw, Melkamu Alemayehu, Daniel Asnake

**Affiliations:** Department of Horticulture, College of Agriculture and Environmental Sciences, Bahir Dar University, P.O. Box 5501, Bahir Dar, Ethiopia

**Keywords:** Fresh leaf yield, Leafy vegetable, Nitrogen nutrient, Paris Island Cos, Phosphorus nutrient, Ecology, Ecology, Environmental sciences, Plant sciences

## Abstract

Lettuce is a popular salad crop and an important cash leafy vegetable, with growing demand in urban and peri-urban areas of Ethiopia. Despite this, research on soil fertility management for lettuce is limited, and productivity remains low. This experiment evaluated the effects of nitrogen and phosphorus fertilizer rates on lettuce growth and yield under irrigation in 2025 at Bahir Dar, Ethiopia. The experiment comprised five nitrogen rates (0, 46, 92, 138, and 184 kg ha^−1^ N) and four phosphorus rates (0, 23, 46, and 92 kg ha^−1^ P_2_O_5_), arranged in a Randomized Complete Block Design with three replications. Data were analyzed using R software. Results revealed that the main effects of nitrogen and phosphorus rates significantly (*P* ≤ 0.001) influenced plant height, number of leaves per plant, and leaf width of lettuce, while leaf length was not affected by phosphorus rates. The interaction of nitrogen and phosphorus significantly (*P* ≤ 0.001) influenced days to maturity, fresh weight of leaf per plant, marketable fresh leaf yield, and total fresh leaf yield. Partial budget analysis revealed that the highest net benefit (241,070 ETB ha^−1^) with a marginal rate of return of 190.51% was achieved from the application of 138 kg ha^−1^ N with 92 kg ha^−1^ P_2_O_5_. This combination was identified as economically optimal and can be recommended for lettuce production in the study area and similar agro-ecological zones. However, the experiment was conducted at a single location and over one season, which may limit generalizability. Varietal differences and environmental impacts were also not assessed; hence, future research should include multi-location, multi-season trials and evaluate varietal differences and environmental impacts.

## Introduction

Lettuce (*Lactuca sativa* L.) is an annual leafy vegetable belonging to the family Asteraceae, the largest of the dicotyledonous families^1^. It is believed to have originated in the Mediterranean region, particularly in Ancient Egypt, where it was initially grown for its oil-rich seeds as early as 4500 B. C^2,3^. It is an economically important salad crop with the highest consumption rate worldwide^4^. It is an excellent source of vitamin A, C, and minerals, including calcium, iron and phosphorus^5^. It is also an important source of phytochemicals^6,7^, lactucin and lactucopicrin compounds^8^. Even though lettuce is an important vegetable in terms of its nutritional value and has a major role in modern urban agriculture, it is one of the neglected and underutilized crops in Ethiopia^9,10^.

Lettuce is produced commercially and in home gardens in many countries^11,12^. According to FAOSTAT^13^, about 28.7 million metric tons of lettuce and chicory are produced on 1.2 million hectares worldwide, with an average productivity of 23.92 t ha^−1^. Kuwait (49.31 t ha^−1^) is the world’s top producer, followed by United States (35.07 t ha^−1^), Hungary (34.85 t ha^−1^), Saudi Arabia (34.82 t ha^−1^), Germany (30.20 t ha^−1^), and China (23.52 t ha^−1^). In Africa, the Democratic Republic of Congo is the leading country, producing 41.33 t ha^−1^, followed by Kenya (27.96 t ha^−1^), Niger (26.75 t ha^−1^), and Egypt (19.75 t ha^−1^). However, Ethiopia produces 0.27 t ha^−1^, which is very low compared with the other countries (FAOSTAT^13^. This is due to poor agronomic practices like irrigation, fertilization, spacing, weeding and disease control^14^.

Fertilizer requirements of a crop, including lettuce depend on various factors including crop type, soil fertility status, environmental conditions as well as method of application^15^. However, proper nutrient management has a significant advantage to the enhancement of production and productivity of lettuce. The prerequisite of crop cultivation is the application of fertilizer at an appropriate time, dose and proper method of application^16,17^. Nitrogen is the most important nutrient for successful cultivation of vegetables, including lettuce. In this regard, Kharade et al.^18^ and Yebirzaf et al.^19^ reported that nitrogen positively affects the diameter of leaves, total number of leaves, and the fresh and dry weight of leaves of lettuce plants. Phosphorus is also required for all crops for the formation and translocation of carbohydrates, the development of root systems and other morphological characters. It enhances maturity and earliness in flowering^20^. Application of phosphorus nutrients significantly enhanced the plant height, number of leaves per plant, leaf length, leaf width, fresh weight as well as yield of lettuce^21,22^. Moreover, combined applications of nitrogen and phosphorus nutrients have a positive significant effect on the growth attributes and fresh leaf yields of lettuce^18,23^. On the other hand, an excessively high amount of nitrogen and phosphorus encourages bolting, which is an undesirable characteristic of lettuce^23^.

The application of optimal nitrogen and phosphorus fertilizer rates is essential for enhancing lettuce production^24,25^. However, in the study area, farmers commonly apply blanket rates of DAP (100–150 kg ha^−1^) and urea (50–100 kg ha^−1^)^26^, yet lettuce yields and productivity in the Amhara Region, including Bahir Dar and its surroundings, remain low. These practices are based on general vegetable recommendations rather than being tailored to the specific nutrient requirements of lettuce or the local soil fertility conditions. Furthermore, there are currently no experimentally validated, site-specific nitrogen and phosphorus fertilizer recommendations and other soil fertility amelioration approaches for irrigated lettuce production in the study area. It was hypothesized that the growth and yield of lettuce would respond significantly to increasing rates of nitrogen and phosphorus under irrigation conditions, with an optimal combined application rate that maximizes both productivity and nutrient use efficiency under the edaphic and climatic conditions of the study area. Accordingly, this study was conducted to evaluate the effects of nitrogen and phosphorus on the growth and yield of lettuce and to determine the optimal fertilizer rates for economically efficient lettuce production, thereby supporting the livelihoods of smallholder farmers in the study area.

## Materials and methods

### Description of the experimental site

The experiment was conducted at Zenzelima Campus of Bahir Dar University on the Horticulture Department experimental site in 2025 under irrigation conditions. The site is geographically located at 11°37’18’' N latitude and 37°27’33’' E longitude with an altitude of about 1921.8 m above sea level. According to the West Amhara Meteorological Service Agency (unpublished), the area received mean annual rainfall of 1248.8 mm and mean minimum and maximum temperatures of 9.7 and 27.5 °C, respectively. During the experimental period (February to April), the mean minimum and maximum temperatures of the area were 12.5 and 30.97 °C, respectively.

### Experimental materials

The Paris Island Cos lettuce variety was used as a test crop. This variety is a romaine lettuce type and its leaves are long with a deep green color. It thrives at moderate temperatures, typically between 15 °C and 20 °C. The variety has a relatively long shelf life and resistant to bolting compared to other lettuce types. The productivity of this variety is 12.5 t ha^−1^. It takes 55 days to mature after transplanting^26^. The seed of the variety was purchased from vegetable seed suppliers in Bahir Dar city. Urea (46% N) and Triple Super Phosphate (TSP) (46% P_2_O_5_) were used as a source of nitrogen and phosphorus, respectively.

### Treatments and experimental design

The treatments consisted of five levels of nitrogen rates (0, 46, 92, 138 and 184 kg ha^−1^ N) and four phosphorus rates (0, 23, 46 and 92 kg ha^−1^ P_2_O_5_) in a 5*4 factorial combination laid out using Randomized Complete Block Design with three replications. Although there are no national fertilizer recommendations for lettuce production, 92 kg ha^−1^ N and 46 kg ha^−1^ P_2_O_5_ were used as a benchmark for this experiment^27^. The total number of plots were 60. Each plot has gross and net plot areas of 2.4 m^2^ and 1.44 m^2^, respectively. Blocks and plots were separated by 1 m and 0.3 m, respectively. A plot accommodated seedlings at a spacing of 40 and 30 cm between rows and seedlings, respectively, were transplanted as recommended by MoANR^26^.

### Experimental soil sampling and analysis

Soil samples were collected before transplanting along the two diagonal lines from nine randomly selected points at a depth of up to 20 cm via an auger. The physicochemical properties such as pH, EC, organic matter, organic carbon, total nitrogen, available phosphorus and textural class of the soils were analyzed at the Soil and Plant Nutrition Laboratory of the College of Agriculture and Environmental Sciences, Bahir Dar University. The results of soil analysis are presented in Table 1. The results of this laboratory examination showed that to support the successful production of lettuce at the study site, nitrogen and phosphorus nutrients must be applied.

**Table 1:** Pre-planting soil physicochemical properties of the experimental site.

Particle size	Values	Rating	Methods	References
Sand (%)	60		Hydrometer	^34^
Silt (%)	28			
Clay (%)	12			
Texture class	Sandy loam			
pH (H_2_O)	6.44	Slightly acidic	Potentiometric	^35^
OM (%)	3.47	Medium		^36^
OC (%)	2.02	Moderate	Walkley-Black Wet Oxidation	^36^
EC (ds/m)	0.017	Low salinity	Conductometric	^37^
TN (%)	0.13	Medium	Kjeldahl Digestion	^38^
AVP (mg/kg)	8.44	Low	Olsen	^39^

pH = power of hydrogen; OM = Organic matter; OC = Organic carbon; EC = Electrical conductivity; TN = Total nitrogen; AVP= Available phosphorus.

### Management of experimental plants and fields

Seeds were sown in the greenhouse on a well-prepared seedbed in February 2025. Seedbeds were covered with fine soil and lightly mulched with dry grass. Regular watering was done using a watering cane and seedlings were thinned out to maintain a healthy and optimal plant population^26^. After three weeks, seedlings had four true leaves and were transplanted to the main field^28^. One day before transplanting, seedbeds were irrigated to enhance easy uprooting and prevention of root damage. The experimental field was cultivated and leveled by man-power and the plots were prepared. During transplanting, only healthy, uniform and vigorous seedlings were transplanted on the prepared plots^26^. Replanting was done within a week after transplanting for dried and unestablished seedlings. The experiment was conducted during the dry (winter) season, when rainfall was absent. Thus, irrigation water was uniformly applied to all experimental plots at two-day intervals using a watering can (15 L holding capacity), ensuring consistent moisture conditions across treatments throughout the experimental period^26^. Irrigation water was sourced from Lake Tana. TSP was applied at the time of transplanting based on the experimental level of phosphorus. Urea was applied into two split doses of equal amounts: the first 50% of urea at time of transplanting and the remaining 50% at two weeks after transplanting^19^. All agronomic practices were applied uniformly according to the recommendation made for lettuce^26^. No remarkable attacks of insect pests and diseases were found in the lettuce field under this study.

### Data collection

#### Phenological parameter of lettuce

*Days to maturity (days)*: Lettuce leaves mature when they are turgid, dark-green, have optimal leaf size and texture as well as the leaves show an elongated upright position depending on the type and variety. Accordingly, days to maturity were recorded by counting the number of days elapsed from the date of transplanting up to the date when 50% of the plants in the plot contained matured leaves as indicated by Gil et al.^29^.

#### Growth parameters of lettuce

*Plant height (cm)*: The plant height of six randomly taken plants grown in the net plot area were measured from the ground level to the tip of the longest leaves at harvest using rulers and the mean values were computed for further analysis as indicated by Yebirzaf et al.^19^ and Bright Affrim-Tiripa^30^]. *Number of leaves per plant (count)*: The total number of leaves of six randomly taken plants grown in the net plot area was counted at harvest stage and the averages were computed and used for analysis as indicated by Islam et al.^23^. *Leaf length (cm)*: The leaf length was measured from the base (end of sheath) to the tip of the leaf from six randomly taken plants grown from the net plot area at harvest using a ruler and the mean values were computed and used for analysis^30^. *Leaf width (cm)*: The leaf width was recorded by measuring the width at the middle part of the leaves (at widest part of the leaves) from six randomly taken plants grown from the net plot area at harvest stage and the averages were computed for further analysis.

#### Yield parameters of lettuce

*Fresh weight of leaves per plant (g)*: The fresh weight of leaves of six randomly taken plants grown in the net plot area at harvest stage were weighed using digital weighing balance and the average value in grams was computed and used for further analysis as indicated by Bright Affrim-Tiripa^30^. *Marketable fresh leaf yield (t ha*^*−1*^*)*: Leaves that are healthy and free from any physiological disorder are considered as marketable^30,31^. Such leaves harvested from the net plot area were measured in kilograms using a digital weighing balance and expressed in tons per hectare. *Unmarketable leaf yield (%)*: Leaves that are diseased, decayed and physiologically damaged are considered as unmarketable^31^. Such leaves harvested from the net plot area were measured in kilograms and expressed as unmarketable leaf yield in percent in relation to the total weight of leaves using the formula indicated below.

$${\mathrm{Unmarketable}}\;{\mathrm{leaf}}\;{\mathrm{yield}}\,\left( \% \right) = \left( {\frac{{Unmarketable\;leave\;weight\,\left( {kg} \right)}}{{Total\;weight\;of\;leaves\,\left( {kg} \right)}}} \right) \times 100$$

*Total fresh leaf yield (t ha*^*−1*^*)*: The total fresh leaf yield was recorded by the summation of marketable and unmarketable leaf yields.

### Statistical analysis

All the collected data were subjected to analysis of variance using R software version 4.1.3. Before conducting ANOVA, normality assumptions were checked using the Shapiro-Wilk test. Mean separation was done using Least Significant Difference (LSD) at 5% level of probability^32^.

### Partial budget analysis

The analysis of the partial budget and marginal rate of return (MRR) was done following the procedures described by CIMMYT^33^. The marketable fresh leaf yield was downscaled by 10% since the experimental yields are usually higher than those of farmers. The gross benefits of the treatments were computed by multiplying the adjusted marketable fresh leaf yield with the farm gate price (15 ETB kg^−1^) of lettuce leaves prevailing in the study area during harvest. The costs of urea and TSP and their costs of application were 36 ETB kg^−1^, 38 ETB kg^−1^ and 150 ETB per man, respectively. Net benefit was calculated by subtracting the total variable costs from the gross benefit of each treatment where the cost of fertilizers and labor required for their placement were considered as variable costs. MRR was calculated as a change of net benefit divided by change of cost in the treatment and expressed in percentage^33^.

$${\mathrm{MRR}}\,\left( \% \right) = \left( {\frac{{Change\;of\;Net\;Benefit}}{{Change\;of\;Total\;Variable\;Cost}}} \right) \times 100$$

## Results and discussion

### Phenological parameters of lettuce

#### Days to maturity

The main effects of nitrogen (*P* ≤ 0.01) and phosphorus (*P* ≤ 0.001) rates, as well as their interaction significantly (*P* ≤ 0.001) influenced the days to maturity of lettuce. As shown in Table 2, the earliest maturity was recorded from a combined application of 184 kg ha^−1^ N with 46 kg ha^−1^ and 92 kg ha^−1^ P2O_5_ and the longest days to maturity were observed on the control treatment. Application of 184 kg ha^−1^ N with 46 and 92 kg ha^−1^ P_2_O_5_ reduced days to maturity by about 26.1% compared to the control plots. This might be due to their synergistic effects, where the availability of phosphorus increases the uptake efficiency of nitrogen and vice versa, thereby boosting protein synthesis, supports energy transfer and early root development. These processes accelerate the accumulation of carbohydrates, which in turn hasten the days to maturity of lettuce. The result is consistent with the findings of Rathter et al.^40^, who clearly indicated that combined application of organic and inorganic fertilizers minimized the number of days required for the maturity of lettuce as compared to the control. Similarly, Simon et al.^41^ also reported that bio-char and NPS accelerated days to the first harvest of Swiss hard.

**Table 2:** Days to maturity of lettuce as influenced by the interaction of nitrogen and phosphorus rates.

Nitrogen rate (kg ha^−1^ *N*)	Phosphorus rate (kg ha^−1^ *P*_2_O_5_)			
	0	23	46	92
0	44.67^a^	40.67^bc^	37.33^def^	34.00^ghi^
46	42.00^b^	37.33^def^	36.00^efg^	33.67^hi^
92	41.00^b^	37.33^def^	34.33^ghi^	33.33^hi^
138	38.67^cd^	35.33^fgh^	34.33^ghi^	33.33^hi^
184	37.67^de^	34.33^ghi^	33.00^i^	33.00^i^
P-value				***
CV (%)				3.76

*** = very highly significant at *P* ≤ 0.001; CV = Coefficient of variation; means followed with the same letter(s) are not significantly different.

### Growth parameters of lettuce

#### Plant height

**Fig. 1:**
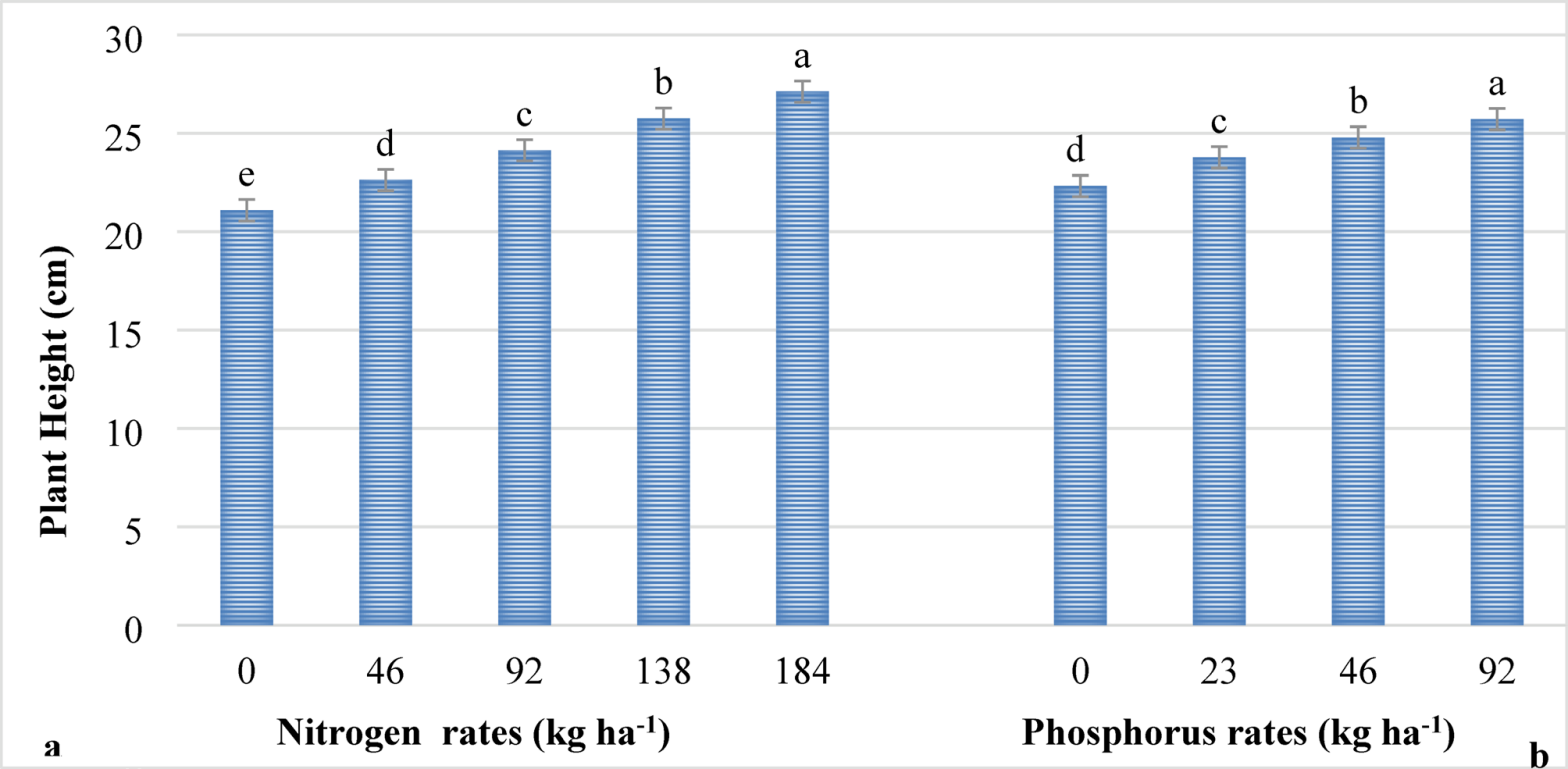
The main effects of nitrogen (**a**) and phosphorus (**b**) rates on plant height. *Note*: Means followed with the same letter(s) are statistically similar at *P* ≤ 0.001.

Nitrogen and phosphorus rates significantly (*P* ≤ 0.001) influenced plant height, while their interaction did not (*P* > 0.05) influence plant height of lettuce. The tallest plants (27.12 cm) were recorded on plots supplied with the highest rate (184 kg ha^−1^ N) of nitrogen, whereas the shortest plant height (21.09 cm) was recorded from lettuce grown without nitrogen fertilizer as indicated in Fig. 1. This might be due to the fact that nitrogen in the form of urea is readily soluble in soil solution and thereby instantly available to vegetative growth of lettuce. Nitrogen is a constituent of chlorophyll that enhances photosynthesis activities, leading to enhanced vigor. It is also vital for the synthesis of amino acids, proteins and enzymes which drive cell division and elongation^42^ that directly increases the plant height of lettuce. The results of this study agree with the findings of different scholars who reported that increased application of nitrogen rates significantly increased the height of lettuce^19,23,43,44^.

Similarly, the highest plant height (25.72 cm) was measured by the application of 92 kg ha^−1^ P_2_O_5_, while the shortest plant (22.32 cm) was recorded on plants without phosphorus application (Fig. 1). This is due to the fact that phosphorus is crucial for early root development and enhances water and nutrient uptake, which directly supports the elongation of plants. Phosphorus fertilizer ensures favorable conditions for cell division and helps in the elongation of lettuce plants with optimum vegetative growth. These results are in line with Kharade et al.^18^, Hong et al.^22^, and Akter et al.^45^, who reported that increased application of phosphorus rates increases the plant height of lettuce.

#### Number of leaves per plant

**Fig. 2:**
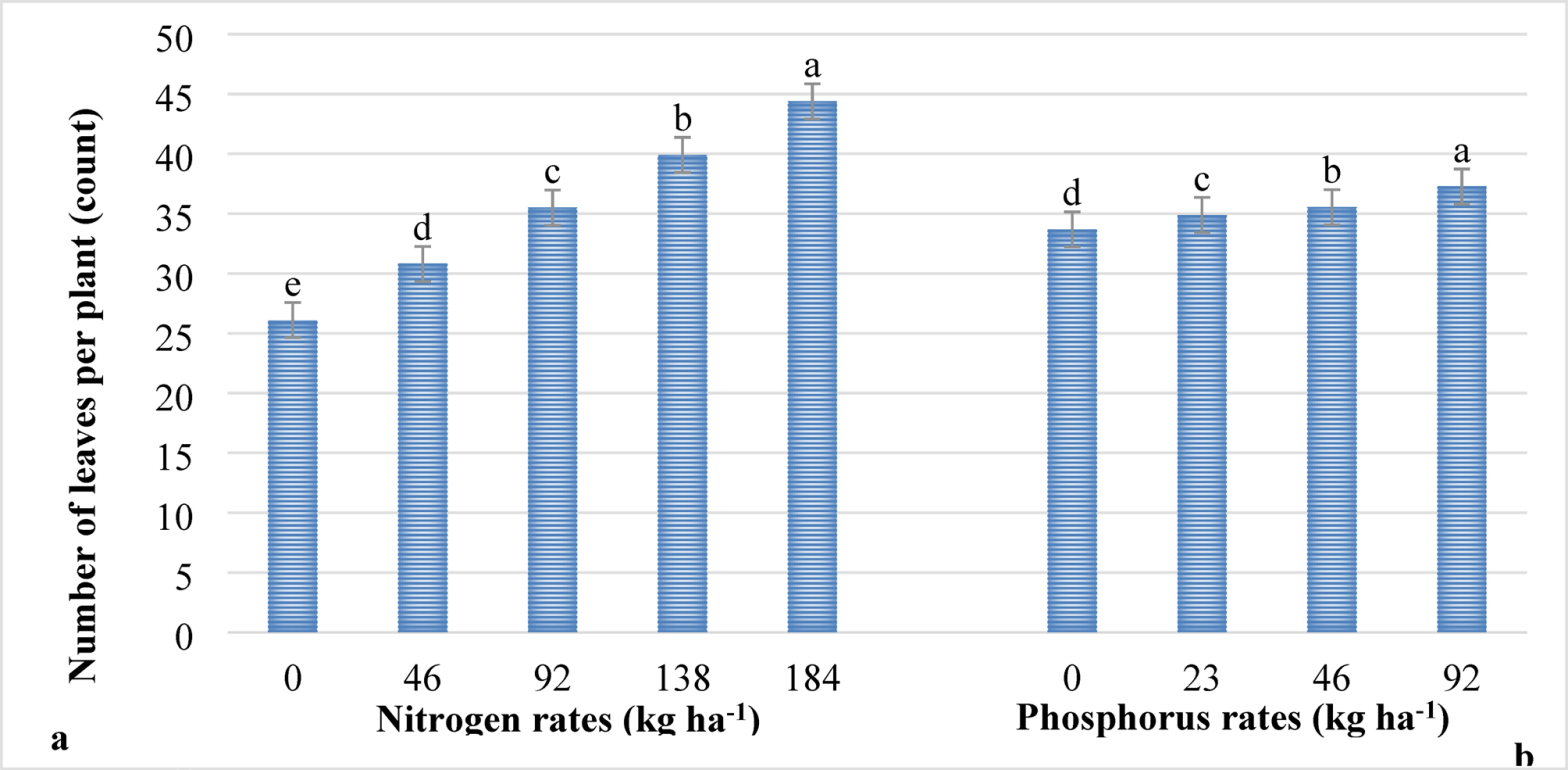
The main effects of nitrogen (**a**) and phosphorus (**b**) rates on the number of leaves per plant of lettuce. *Note*: Means followed with the same letter(s) within the group are not significantly different at *P* ≤ 0.001.

The number of leaves per plant was significantly (*P* ≤ 0.001) affected by the main effects of nitrogen and phosphorus fertilizer rates. On the other hand, the interaction effect of both factors did not influence (*P* > 0.05) the number of leaves per plant of lettuce. The highest number of leaves per plant (44.39) was recorded from the highest rate of nitrogen (184 kg ha^−1^ N), while the lowest number of leaves per plant (26.10) was recorded without nitrogen application as indicated in Fig. 2. Similarly, the highest number of leaves per plant (37.26) was recorded from the highest rate of phosphorus (92 kg ha^−1^ P_2_O_5_), while the lowest number of leaves per plant (33.68) was recorded on lettuce without phosphorus application (Fig. 2).

Nitrogen and phosphorus play an important role in the growth and development of lettuce. It could be associated with the production of more food materials through the process of photosynthesis. Similarly, phosphorus promotes development of roots and cell division, which in turn drives the formation of a greater number of leaves. Results of the present study are in agreement with the findings of other researchers where the application of nitrogen and phosphorus promoted the number of leaves of lettuce^19,43–45^. In the research conducted by Tsigereda et al.^27^, the application of chemical fertilizers in lettuce boosted the number of leaves per plant significantly by 25.8% over the control treatments. Similarly, Simon et al.^41^ reported that the increasing rate of NPS fertilizer as well as bio-char generally increased the number of leaves per plant of Swiss chard.

#### Leaf length and width

**Fig. 3:**
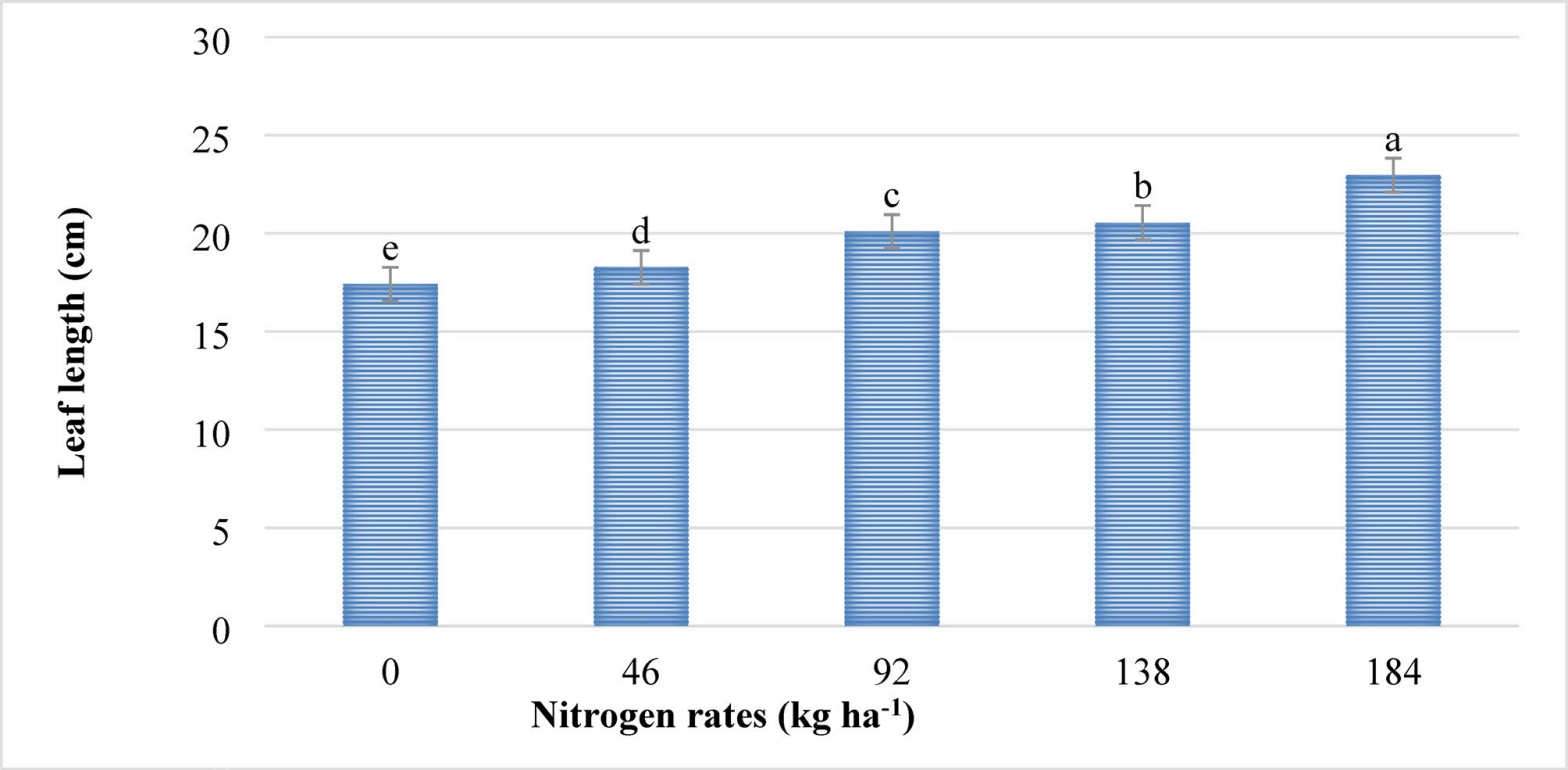
The main effect of nitrogen rates on the leaf length (cm) of lettuce. *Note*: Means followed with the same letter(s) are statistically similar at *P* ≤ 0.001.

**Fig. 4:**
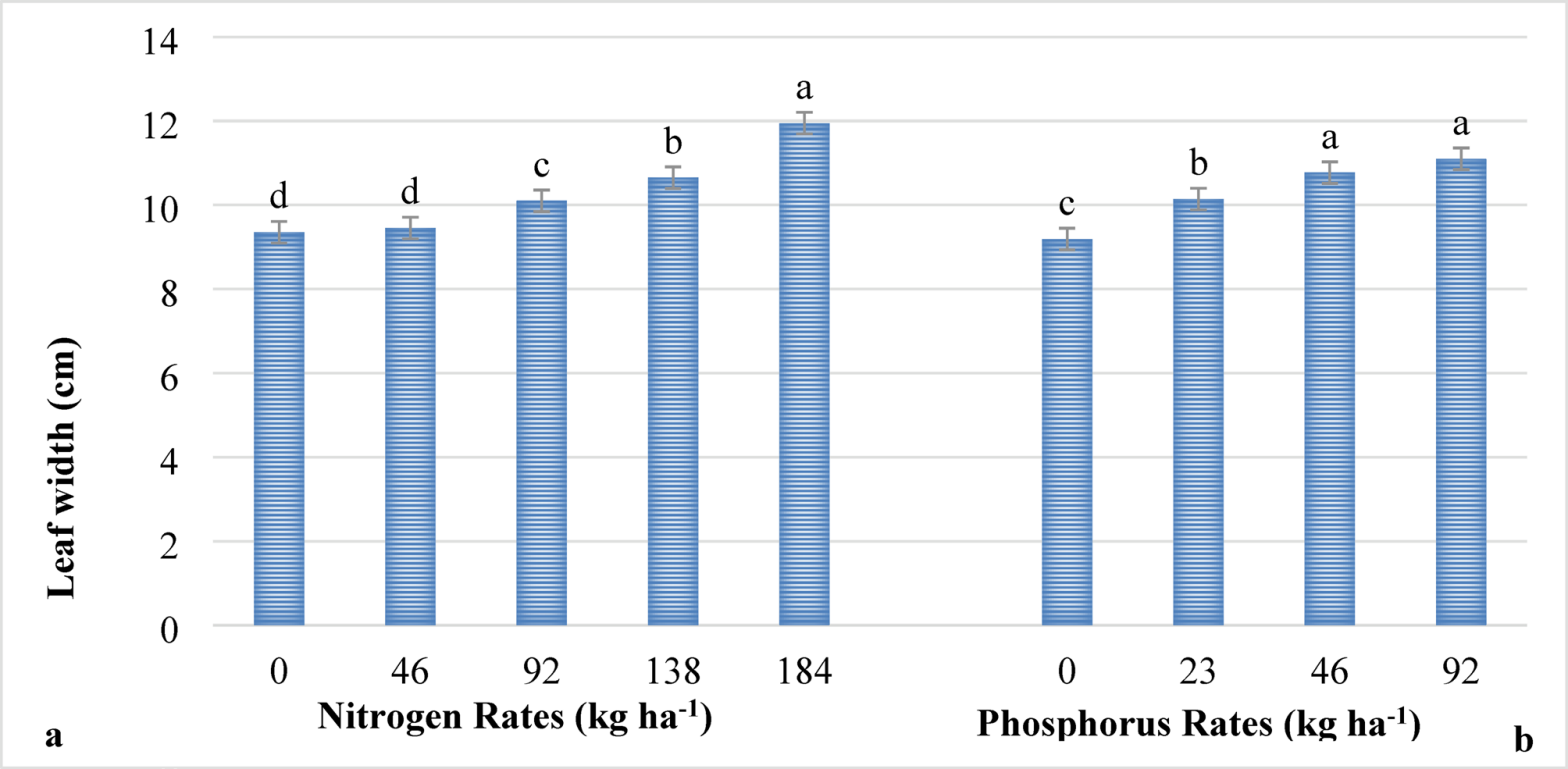
The main effects of nitrogen (**a**) and phosphorus (**b**) rates on the leaf width (cm) of lettuce. *Note*: Means followed with the same letter(s) are statistically similar at *P* ≤ 0.001.

The leaf length of lettuce was significantly (*P* ≤ 0.001) influenced by the main effect of nitrogen. The leaf width, on the other hand, was influenced (*P* ≤ 0.001) by the main effect of both nitrogen and phosphorus fertilizers. However, the main effect of phosphorus on leaf length and its interaction with nitrogen did not influence (*P* > 0.05) the length and width of lettuce leaves. The highest leaf length (22.96 cm) as indicated in Fig. 3, and width (11.95 cm) as indicated in Fig. 4 were recorded from plants supplied at the highest rate of nitrogen (184 kg ha^−1^ N), while the shortest leaf length (17.41 cm) (Fig. 3) and narrowest leaves (9.35 cm) (Fig. 4) were recorded from plots where no nitrogen fertilizer was applied. This could be due to the fact that nitrogen is a key component of chlorophyll, which drives the photosynthesis activities that directly contribute to boosting the vegetative growth, including the length and width of lettuce leaves. These results agree with the findings of other researchers where the application of nitrogen fertilizer increased the length^23,43,46,47^ and width Tiru et al.^48^ of lettuce and other similar crops.

Regarding phosphorus fertilizer, the widest leaf width (11.1 cm) was recorded from plants supplied with 92 kg ha^−1^ P_2_O_5_. However, the shortest leaf width (9.19 cm) was recorded from plants without phosphorus application (Fig. 4). This might be due to the fact that phosphorus enhances root development and facilitates enzyme activity and metabolic functions, which improve the nutrient and water uptakes, leading to improved growth and development of leaves and overall plant vigor. Generally, phosphorus plays a vital role in several physiological and biochemical processes that support vegetative growth in lettuce plants. These results are consistent with the findings of Islam et al.^23^ in which the leaf width was enhanced by the application of phosphorus.

### Effects of nitrogen and phosphorus fertilizer rates on yield parameters of lettuce

#### Fresh weight of leaves per plant

The main effects, as well as the interaction effects of nitrogen and phosphorus significantly (*P* ≤ 0.001) influenced the fresh weight of lettuce leaves. Increasing rates of nitrogen as well as phosphorus fertilizers generally increased the fresh weight of leaves per lettuce plant in the present study. The highest fresh weight of leaves per plant was recorded by combined application of 184 kg ha^−1^ N and 92 kg ha^−1^ P_2_O_5_. However, lettuce plants grown without any fertilizer produced the lowest (150.67 g) fresh weight of leaves per plant (Table 3).

A combination of appropriate rates of nitrogen and phosphorus fertilizer is necessary for successful production of vegetable crops, including lettuce. The synergistic effect of nitrogen and phosphorus enhances nutrient uptake, nutrient utilization efficiency and also promotes cell expansion and photosynthetic capacities, which in turn contribute to the increased leaf biomass of lettuce as indicated in the present study. Similar results were also reported by various scholars, who stated that combined application of nitrogen and phosphorus fertilizer at different rates has a positive significant effect on the fresh weight of leaves per plant in lettuce plants^18,22,23,45^. Similarly, Patel et al.^46^ reported that the application of nitrogen and phosphorus fertilizer improves the fresh weight of leaves of spinach plants.

#### Marketable fresh leaf yield

The main effects of nitrogen and phosphorus fertilizer, as well as their interaction significantly (*P* ≤ 0.001) influenced the marketable fresh leaf yield of lettuce. The highest marketable fresh leaf yield was obtained from the combined application of 184 kg ha^−1^ N and 92 kg ha^−1^ P_2_O_5_. Whereas, the lowest marketable fresh leaf yield was recorded from plants grown without the application of nitrogen and phosphorus fertilizers. The combined application of 184 kg ha^−1^ N and 92 kg ha^−1^ P_2_O_5_ increased the marketable fresh leaf yield of lettuce by about 62.34% compared to the control treatment (Table 3).

This might be attributed to the synergistic effects of nitrogen and phosphorus nutrients in improving vegetative growth by enhancing the chlorophyll synthesis and protein formation and nutrient uptake efficiency, which in turn increases healthy and attractive leaves which are acceptable for markets. Similar results were found by Hasan et al.^49^ and Islam et al.^23^ where the interaction effect of nitrogen and phosphorus fertilizer rates significantly influenced the marketable fresh leaf yield of lettuce. The results of the current study are also supported by Tsigereda et al.^27^, who demonstrated that applying inorganic fertilizers and bio-slurry increased the yield of lettuce. Similarly, Singh and Singh^50^ in spinach and Miceli and Miceli^31^ in Swiss chard recorded the highest yield for the combined application of nitrogen and phosphorus fertilizer.

#### Unmarketable leaf yield

The main effects and interaction effects of nitrogen and phosphorus fertilizer significantly (*P* ≤ 0.001) influenced the unmarketable leaf yield of lettuce. The highest unmarketable leaf yield percentage was recorded from the nil application of nitrogen and phosphorus fertilizers, while the least unmarketable leaf yield percentage (6.53%) was recorded from the combined application of 184 kg ha^−1^ N and 92 kg ha^−1^ P_2_O_5_ (Table 3). The highest unmarketable leaf yield percentage was recorded, which could be due to insufficient soil nutrients to support the growth and development of lettuce in the experimental site in the present study (Table 1). Such insufficient nutrients may lead to physiological disorders, uneven leaf size and color, decay, and unattractive leaves of lettuce that in turn increase the unmarketable yield. These results are in line with the findings of Islam et al.^23^, who indicated that the highest unmarketable leaf yield was produced in the nil application of nitrogen and phosphorus fertilizers in lettuce. However, the present results are not in conformity with the findings of Simon et al.^41^, who reported that increasing rates of NPS fertilizer increased unmarketable leaf yields of Swiss chard.

#### Total fresh leaf yield

The analysis of variance showed that nitrogen and phosphorus in their main effects as well as in their interaction effects (*P* ≤ 0.001) influenced total fresh leaf yield of lettuce. As presented in Table 3, the highest total fresh leaf yield was recorded from the combination of 184 kg ha^−1^ N and 92 kg ha^−1^ P_2_O_5_, whereas the lowest total fresh leaf yield was recorded from lettuce plants grown without the application of nitrogen and phosphorus fertilizers. This might be due to the fact that the combined application of nitrogen and phosphorus fertilizer increases the photosynthetic efficiency, nutrient use efficiency, and biomass production. As indicated in the present study, increasing the rate of both macronutrients increased the vegetative growth parameters, leading to the highest total fresh leaf yield of lettuce.

The present result is in line with the findings of previous research where the combined application of nitrogen and phosphorus fertilizers increased the total fresh leaf yield of lettuce^18,22–24,27,45^. Similarly, Yebirzaf et al.^19^ indicated that combined application of nitrogen and farmyard manure progressively increased the total fresh leaf yield of lettuce.

**Table 3:** Interaction effects of nitrogen and phosphorus rates on yield parameters of lettuce.

*N* (kg ha^−1^)	*P* (kg ha^−1^)	Fresh weight of leaf per plant (g)	Marketable fresh leaf yield (t ha^−1^)	Unmarketable leaf yield (%)	Total fresh leaf yield (t ha^−1^)
0	0	150.67^h^	7.37^m^	21.23^a^	8.60^p^
	23	226.12^g^	12.54^k^	17.93^c^	13.40^n^
	46	250.36^ef^	13.09^j^	15.46^e^	14.10^l^
	92	256.09^ef^	14.70^h^	14.10^g^	14.93^k^
46	0	224.23^g^	11.54^l^	19.10^b^	12.93^o^
	23	247.19^ef^	15.28^g^	14.96^f^	15.50^j^
	46	275.39^d^	16.23^e^	11.90^i^	17.00^h^
	92	319.19^ab^	16.68^e^	10.76^k^	17.33^g^
92	0	230.61^g^	12.50^k^	17.73^c^	13.86^m^
	23	255.63^ef^	15.76^f^	13.16^h^	16.30^i^
	46	283.51^cd^	17.24^d^	9.96^m^	17.90^f^
	92	321.14^ab^	17.77^c^	8.83^n^	18.70^d^
138	0	245.69^f^	13.86^i^	16.36^d^	15.10^k^
	23	292.60^c^	16.38^e^	11.36^j^	17.33^g^
	46	316.79^b^	18.47^b^	8.83^n^	18.90^c^
	92	327.97^ab^	19.32^a^	7.40^p^	19.76^b^
184	0	258.35^e^	13.97^i^	15.70^e^	15.60^j^
	23	292.60^c^	17.46^cd^	10.46^l^	18.26^e^
	46	322.88^ab^	18.47^b^	8.03^o^	19.60^b^
	92	330.59^a^	19.57^a^	6.53^q^	20.90^a^
P-value		***	***	***	***
CV (%)		2.72	12.42	13.21	9.33

*** = very highly significant at *P* ≤ 0.001; CV = Coefficient of variation; means followed with the same letter(s) are not significantly different.

### Partial budget analysis

The partial budget analysis indicated that the combined application of 138 kg ha^−1^ N with 92 kg ha^−1^ P_2_O_5_ resulted in high net benefits with an acceptable MRR of 190.51%. The 190.51% MRR indicates that investing one ETB in the treatment combination of 138 kg ha^−1^ N and 92 kg ha^−1^ P_2_O_5_ enables the growers to obtain a return of 1.90 ETB ha^−1^. However, the lowest net benefit (99,495 ETB ha^−1^) was obtained from the nil application of nitrogen and phosphorus nutrients (Table 4). As presented in Table 4, an additional increase in the combined application of nitrogen and phosphorus rate beyond 138 kg ha^−1^ N with 92 kg ha^−1^ P_2_O_5_ reduced the net return of lettuce.

**Table 4:** Partial budget analysis for lettuce production as influenced by rates of nitrogen and phosphorus fertilizers.

Treatments (kg ha^−1^)	MLY (t ha^−1^)	AdMLY (t ha^−1^)	TVC (ETB ha^−1^)	NB (ETB ha^−1^)	Dominance analysis	MRR (%)
0 × 0	7.37	6.63	0	99,495		
0 × 23	12.54	11.29	2,200	167,090		3,072.50
46 × 0	11.54	10.39	4,050	151,740	D	
0 × 46	13.09	11.78	4,250	172,465		262.20
46 × 23	15.28	13.75	6,250	200,030		1,378.25
92 × 0	12.50	11.25	7,800	160,950	D	
0 × 92	14.70	13.23	8,200	190,250	D	
46 × 92	16.68	15.01	8,300	216,880		821.95
92 × 23	15.76	14.18	10,000	202,760	D	
138 × 0	13.86	12.47	11,550	175,560	D	
92 × 46	17.24	15.52	12,050	220,690		101.60
46 × 46	16.23	14.61	12,250	206,855	D	
138 × 23	16.38	14.74	13,750	207,380	D	
184 × 0	13.97	12.57	15,300	173,295	D	
138 × 46	18.47	16.62	15,800	233,545		342.80
92 × 92	17.77	15.99	16,000	223,895	D	
184 × 23	17.46	15.71	17,500	218,210	D	
184 × 46	18.47	16.62	19,550	229,795	D	
138 × 92	19.32	17.39	19,750	241,070		190.51
184 × 92	19.57	17.61	23,500	240,695	D	

MLY = marketable leaf yield; AdMLY = Adjusted marketable leaf yield; TVC = Total variable cost; NB = Net benefit; MRR = Marginal Rate of Return; D = indicates a dominated treatment, meaning higher cost with lower or equal net benefit compared to a preceding treatment.

## Conclusion

The results of the present study revealed that increasing the nitrogen and phosphorus nutrient levels boosted the key growth characteristics of lettuce, including the days to maturity, plant height, number of leaves per plant, leaf length, and leaf width. Similarly, the interaction effects of the two factors influenced the yield parameters of lettuce, including fresh weight of leaves per plant, marketable fresh leaf yield, unmarketable leaf yield, and total fresh leaf yield. The combination of 138 kg ha^−1^ N and 92 kg ha^−1^ P_2_O_5_ was found to be economically optimal and provides a preliminary recommendation for the production of lettuce in the study area and in areas with similar agro-ecological conditions, as it resulted in the highest net benefit (241,070 ETB ha^−1^) with an acceptable marginal rate of return (190.51%). However, due to time and resource limitations, the study was conducted during one growing season at a single location. Similarly, potassium was not analyzed because soils in the Amhara region are generally rich in potassium and also due to laboratory constraints. In addition, varietal differences in nutrient use efficiency and the potential environmental implications associated with nitrogen and phosphorus application, such as nutrient leaching and their effects on soil and water quality, were not assessed in the present experiment. Future research should therefore include multi-season and multi-location trials, evaluation of different lettuce varieties, and assessment of the environmental impacts of nitrogen and phosphorus fertilization in order to validate and refine these findings for wider adoption.

## Data Availability

The data that support the findings of this study are available upon reasonable request.
